# Aluminum Alleviation of Iron Deficiency Chlorosis Is Conserved in Wild Rice Relative *Oryza rufipogon* and in Maize

**DOI:** 10.3390/plants15010159

**Published:** 2026-01-05

**Authors:** Jover da Silva Alves, Yugo Lima-Melo, Andriele Wairich, Vic Martini Sasso, Vitor L. Nascimento, Raul Antonio Sperotto, Luciane Almeri Tabaldi, Gustavo Brunetto, Felipe Klein Ricachenevsky

**Affiliations:** 1 Biotechnology Center, Federal University of Rio Grande do Sul, Porto Alegre 91501-970, RS, Brazil; joversalves@gmail.com (J.d.S.A.); yugo_lima@yahoo.com.br (Y.L.-M.); andriwairich@gmail.com (A.W.); 2 Botany Department, Federal University of Santa Maria, Santa Maria 97105-900, RS, Brazil; vicksasso@yahoo.com.br (V.M.S.); luciane.tabaldi@ufsm.br (L.A.T.); 3 Biology Department, Natural Sciences Institute, Federal University of Lavras, Lavras 37200-900, MG, Brazil; vitor.nascimento@ufla.br; 4 Graduate Program in Plant Physiology, Botany Department, Biology Institute, Federal University of Pelotas, Pelotas 96160-000, RS, Brazil; raulsperotto@gmail.com; 5 Soil Department, Federal University of Santa Maria, Santa Maria 97105-900, RS, Brazil; brunetto.gustavo@gmail.com; 6 Botany Department, Federal University of Rio Grande do Sul, Porto Alegre 91501-970, RS, Brazil

**Keywords:** iron deficiency, aluminum, chlorosis, maize, rice, *Oryza rufipogon*

## Abstract

Aluminum (Al), an element that has no biological function described in plants, is commonly found in acidic soils, reducing plant growth, despite some beneficial effects reported in the literature. Iron (Fe) is an essential nutrient for plants, and Fe deficiency causes leaf interveinal chlorosis. Remarkably, rice (*Oryza sativa*), a C_3_ crop considered tolerant to Al, shows alleviation of Fe deficiency chlorosis when exposed to Al, suggesting that Al can positively impact Fe homeostasis. However, whether this effect is observed only in rice or is common to other plant species is unknown. The rice wild progenitor *Oryza rufipogon* is closely related to the domesticated species, sharing several traits such as a semi-aquatic habit and use of the combined strategy for Fe uptake. Maize (*Zea mays*), on the other hand, is a C_4_ plant, adapted to well-aerated soils, and uses a classic chelation-based strategy for Fe uptake. Here we used these two Poaceae representatives to determine whether Al excess could alleviate Fe deficiency chlorosis in species other than rice. Although Al caused toxicity irrespective of Fe levels, its addition essentially abolished chlorosis in Fe-deficient plants. The expression of Fe deficiency-induced marker genes was reduced to control levels in both species, suggesting that the Al alleviation effect leads to systemic signaling and down-regulation of Fe uptake mechanisms. Al alleviation partially rescued photosynthetic machinery inhibited by Fe deficiency, suggesting that leaves are maintaining photosynthetic activity when Al is present even under low Fe conditions. Taken together, our data show that the Al alleviation effect is shared by two other Poaceae species in addition to *O. sativa* and suggest that it might not be directly linked to domestication, changes in C_3_/C_4_ metabolism, or Al tolerance levels found in different species.

## 1. Introduction

Cultivated rice (*Oryza sativa*), a Poaceae family member, is one of the most important domesticated crops, serving as a model species for cereal crops. With an annual global production of approximately 800 million tons [1], rice is a staple food for over half of the world’s population, meeting the caloric demands driven by rapid population growth [2]. *Oryza sativa*, a C_3_ photosynthetic plant, is part of the *Oryza* genus, which consists of 27 species with 11 different genome types, comprising 17 diploid and 10 allotetraploid genomes varying in size by up to 3.4-fold. Such high variation in genome size and ploidy represents approximately 15 million years of evolution [2,3]. *Oryza rufipogon*, the wild progenitor of *O. sativa* ssp. *japonica* [4], is a perennial plant able to grow in swamps, channels, marshes, and boundaries of ponds and lakes [5]. As with other wild rice species of the *Oryza* genus, *O. rufipogon* represents a valuable gene pool used to broaden the genetic diversity of cultivated rice. It has been used to search for tolerance to both biotic and abiotic stresses, including salinity stress, aluminum (Al) tolerance, and phosphorus (P) deficiency [6,7].

Another species of the Poaceae family, maize (*Zea mays*) is an important C_4_ photosynthetic cereal, ranking as one of the most widely grown crop worldwide, along with wheat and rice [8]. With an annual cultivation area of approximately 197 million hectares, maize is a staple food providing over 20% of the total caloric intake in numerous countries [9,10]. Its role extends beyond direct human consumption, contributing to livestock production and as a raw material in various industries, such as biofuel and ethanol production, highlighting its versatility and essential role in global food security [11,12,13]. Due to its large cultivation area, maize is grown in a wide variety of soils, exposing the plants to soil types that can result in complex stresses related to mineral disorders. Considering that the ionome is highly integrated, and plants have several mechanisms to control relative concentrations of nutrients [14,15,16], a deficiency or excess in one element can drastically impact the concentration or the homeostasis of others [14,15,17,18].

Approximately 40–50% of the world’s potentially arable land consists of acidic soils, where Al toxicity is a major factor limiting crop productivity [19,20]. Its soluble ionic form (Al^3+^) inhibits root elongation and reduces absorption of water and essential nutrients, leading to stunted root growth [21,22,23]. Al-sensitive crops such as maize can experience growth impairment and decreased photosynthesis even at low Al concentrations [24,25]. Al toxicity triggers a reduction in leaf expansion, stomatal closure, and photosynthetic efficiency, further impairing plant transpiration [26,27,28]. Strategies to mitigate these effects include breeding Al-tolerant cultivars, applying lime to neutralize soil acidity, and adopting sustainable soil management practices to improve soil quality [29,30]. Nevertheless, given the widespread occurrence of acidic soils, Al toxicity continues to be a major limitation in agricultural production.

Conversely, although iron (Fe) is the fourth most abundant element, it is frequently deficient in soils. It is estimated that one third of Earth’s soils are Fe-deficient [31]. The availability and solubility of Fe in soils are influenced by various factors, including soil pH, redox potential, microbial activity, organic matter content, and soil aeration [32,33,34]. Fe is an essential nutrient for plants, being involved in central functions such as electron transfer during photosynthesis and respiration, as well as in the metabolism of nitrogen (N) and sulfur (S) and chlorophyll biosynthesis [35,36,37]. Due to that, Fe deficiency causes interveinal chlorosis (a hallmark symptom) mainly in young leaves, which is used to diagnose Fe deficiency in field conditions [31,35,36].

Plants have evolved specific mechanisms for Fe uptake: (i) Strategy I, or reduction strategy, common to all non-Poaceae species, and (ii) Strategy II, or chelation strategy, common to Poaceae [16,38]. In the chelation strategy, Fe-deficient plants produce and release Fe(III)-chelating molecules named phytosiderophores (PS), which form highly stable hexadentate Fe(III)-PS complexes that are taken up by root cells via Yellow Stripe-Like (YSL) transporters localized at the plasma membrane [39,40]. In maize, the *ZmYS1* (*Yellow Stripe 1*) gene plays a crucial role in the efficient uptake of Fe(III)-PS complexes. Maize plants lacking this functional ZmYS1 transporter (*zmys1* mutants) exhibit interveinal chlorosis even when Fe is available in the soil, highlighting the importance of this mechanism for Fe acquisition [41]. Moreover, several genes involved in the Fe deficiency response are already characterized in Poaceae species, and their expression levels can be used as markers of Fe-deficient status [42,43,44,45].

Fe homeostasis can interact with the homeostasis of other nutrients, and plants regulate the relative concentrations of elements to maintain balance [16]. Interestingly, recent evidence has shown that Fe deficiency can be mitigated by combined deficiency or excess of other nutrients. For example, while plants under Fe deficiency alone typically develop chlorosis, plants under combined Fe and P deficiencies remain green [46]. A similar response has been observed in rice, barley, tomato, *Lemma gibba*, and cyanobacteria [47,48,49,50], suggesting a conserved mechanism involving Fe–P interaction. Recent studies also showed that Al may influence this balance, as rice plants exposed to combined Fe deficiency and Al excess have decreased Fe deficiency chlorosis and downregulation of Fe deficiency marker genes, which suggests that Al might induce P deficiency, mimicking the same chlorosis-alleviating phenotype [51]. However, since cultivated rice is an Al-tolerant crop [19], it remains unclear whether the same mechanism applies to other Poaceae species.

To test whether Fe deficiency alleviation by Al excess is conserved in other plant species besides rice, we first extended the investigation to *O. rufipogon*, the wild ancestor of cultivated rice [3,5,52]. We found that *O. rufipogon* responded quite similarly to rice, suggesting that the alleviation effect is based on an ancestral mechanism, not related to domestication. Next, we tested whether maize, a domesticated C_4_ and Al-sensitive crop, would also recover from chlorosis when exposed to combined Fe deficiency and Al. We found that Al excess also alleviated Fe deficiency chlorosis in maize. Our data support the hypothesis that the mitigation of Fe deficiency by Al is a conserved phenomenon, observed both in a close wild relative of rice and in maize. This suggests that the mechanism is unrelated to domestication or differences between C_3_ and C_4_ photosynthetic metabolisms and may be conserved across Poaceae, with potential relevance for improving nutrient management in cereal crops grown in acidic soils.

## 2. Results

### 2.1. Al Excess and Fe Deficiency Reduce Root Elongation in Oryza rufipogon

**Figure 1:**
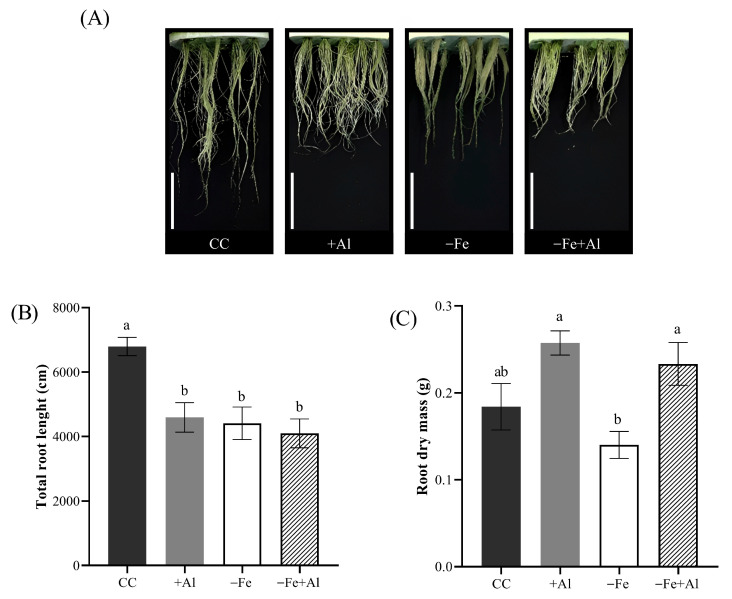
**Root phenotype of *Oryza rufipogon* plants under Fe deficiency and Al excess.** (**A**) Representative images of root systems after ten days of treatment with control solution (CC), 300 µM AlCl_3_ (+Al), no Fe (−Fe), or combined (−Fe+Al). (**B**) Total root length and (**C**) root dry mass of plants shown in (**A**). Different letters indicate statistically significant differences according to Tukey’s test (*p* ≤ 0.05). Values represent mean ± SE (*n* = 5). Scale bar in (**A**) = 15 cm.

To evaluate the effects of Al excess and Fe deficiency on root development in *O. rufipogon*, we analyzed plants grown under four conditions: control (CC in figures), Al excess (+Al; 300 µM AlCl_3_), Fe deficiency (−Fe; no Fe added), and combined Fe deficiency and Al excess (−Fe+Al). Root length was significantly reduced in the +Al, −Fe, and −Fe+Al treatments compared to the control (Figure 1A,B), indicating that both Al excess and Fe deficiency inhibited root elongation, but their combined effects are not additive. This suggests that both Al excess and Fe deficiency negatively affect root growth in *O. rufipogon*, whereas their combination is not additionally toxic. The root dry mass was lower in −Fe plants compared with the control, whereas the +Al and −Fe+Al treatments did not differ from the control (Figure 1C). Together, these findings indicate that Al excess and Fe deficiency independently limit root elongation in *O. rufipogon*.

### 2.2. Al Excess Limits Shoot Development in Oryza rufipogon Independently of Fe

**Figure 2:**
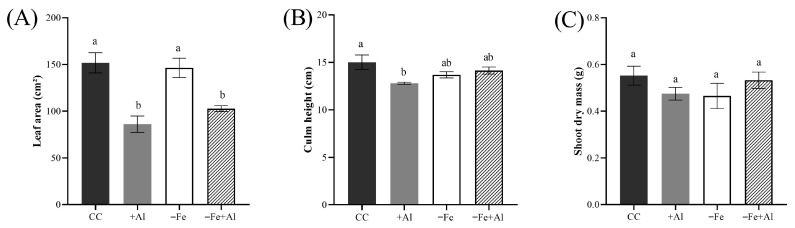
**Shoot phenotype of *Oryza rufipogon* plants under Fe deficiency and Al excess.** (**A**) Leaf area, (**B**) culm height, and (**C**) shoot dry mass of plants treated with control solution (CC), 300 µM AlCl_3_ (+Al), no Fe (−Fe), or combined (−Fe+Al). Different letters indicate statistically significant differences according to Tukey’s test (*p* ≤ 0.05). Values represent mean ± SE (*n* = 5).

Plants under +Al and −Fe+Al showed a reduced leaf area compared to the control and −Fe treatments (Figure 2A). In contrast, plants exposed to −Fe alone maintained a leaf area similar to the control, suggesting that Al excess negatively affects leaf expansion independently of Fe status. Regarding culm height, only the +Al treatment showed lower values compared with the control (Figure 2B). In contrast, the shoot dry mass was similar across all treatments (Figure 2C). These results show that *O. rufipogon* is able to sustain shoot biomass under both Fe deficiency and combined Al excess and Fe deficiency, even though the leaf area is affected.

### 2.3. Al Excess Alleviates Fe Deficiency Chlorosis in Oryza rufipogon

**Figure 3:**
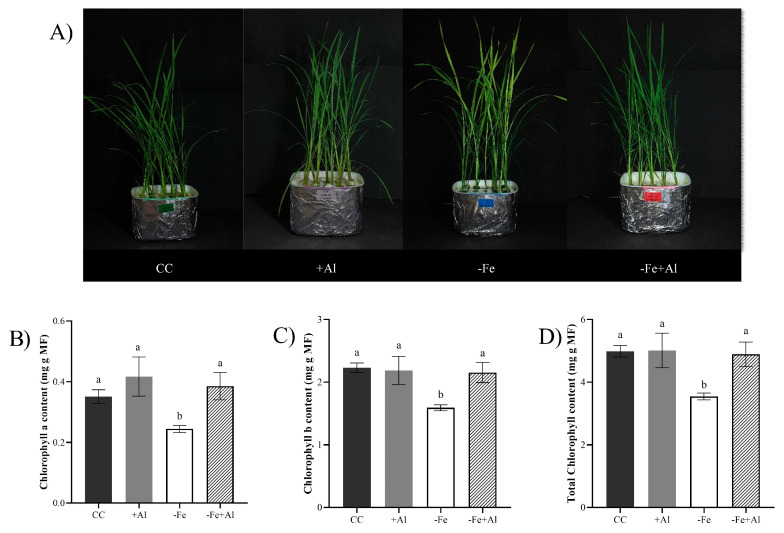
**Visual phenotype and photosynthetic pigment contents in *Oryza rufipogon* plants under Fe deficiency and Al excess.** (**A**) Representative images of the youngest fully expanded leaves after 21 days of treatment. (**B**) Chlorophyll a, (**C**) chlorophyll b, and (**D**) total chlorophyll content in leaves of plants treated with control solution (CC), 300 µM AlCl_3_ (+Al), no Fe (−Fe), or combined (−Fe+Al). Different letters indicate statistically significant differences according to Tukey’s test (*p* ≤ 0.05). Values represent mean ± SE (*n* = 4).

To evaluate whether the alleviating effect of Al on chlorosis observed in cultivated rice (*O. sativa*) [51] is conserved in its ancestral species *O. rufipogon*, we analyzed leaf chlorosis after 21 days of treatment. Plants exposed to +Al alone did not exhibit any visible phenotypic alterations, whereas those under Fe deficiency displayed pronounced chlorosis in the youngest leaf (Figure 3A). However, combined −Fe+Al showed chlorosis alleviation (Figure 3A). For chlorophyll *a*, there was a tendency for decrease in −Fe compared to the control, while both -Al and combined −Fe+Al were higher compared to −Fe (Figure 3B). Chlorophyll *b* and total chlorophyll were both reduced under Fe deficiency, while plants exposed to combined −Fe+Al exhibited alleviated chlorosis (Figure 3B–D). These findings confirm that the chlorosis-alleviating effect of Al excess observed in cultivated rice is also present in *O. rufipogon*.

### 2.4. Al Excess Decreases the Expression of Fe Deficiency Marker Genes in Oryza rufipogon Roots

**Figure 4:**
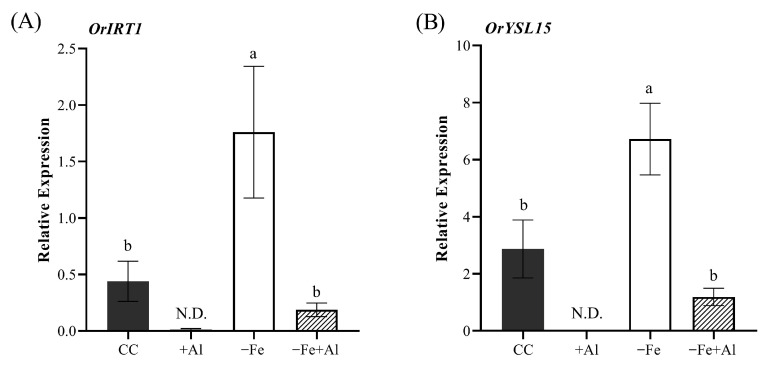
**Relative expression of Fe homeostasis genes in *Oryza rufipogon* roots.** Relative expression of (**A**) *OrIRT1* and (**B**) *OrYSL15* in roots of plants treated with control solution (CC), 300 µM AlCl_3_ (+Al), no Fe (−Fe), or combined (−Fe+Al) after ten days of treatment. Data were normalized to *OrUBQ5*. N.D.: not detected. Different letters indicate statistically significant differences according to Tukey’s test (*p* ≤ 0.05). Values represent mean ± SE (*n* = 4).

To investigate whether the chlorosis-alleviating effect of Al is associated with suppression of the Fe deficiency response, we analyzed the expression of *OrIRT1* (Figure 4A) and *OrYSL15* (Figure 4B) genes in roots of *O. rufipogon* after 10 days of treatment. These genes encode Fe transporters and have previously been used as molecular markers of Fe deficiency in *Oryza* species [43]. As expected, Fe deficiency induced the expression of both genes compared to control plants (Figure 4A,B). In contrast, expression in −Fe+Al plants was similar to control levels, indicating that Al inhibits the transcriptional activation of these Fe-deficiency markers genes.

In plants treated with +Al alone, transcript levels of both genes were not detected, while control plants exhibited basal expression (Figure 4A,B). This pattern suggests that Al may actively repress the expression of Fe deficiency marker genes even under Fe-sufficient conditions, as previously observed in *O. sativa* [51]. Altogether, these data indicate that Al interferes with the Fe signaling pathway and that this regulatory effect is conserved in *O. rufipogon*.

### 2.5. Al Excess Reduces Maize Root Length Independently of Fe Deficiency Without Affecting Root Biomass

**Figure 5:**
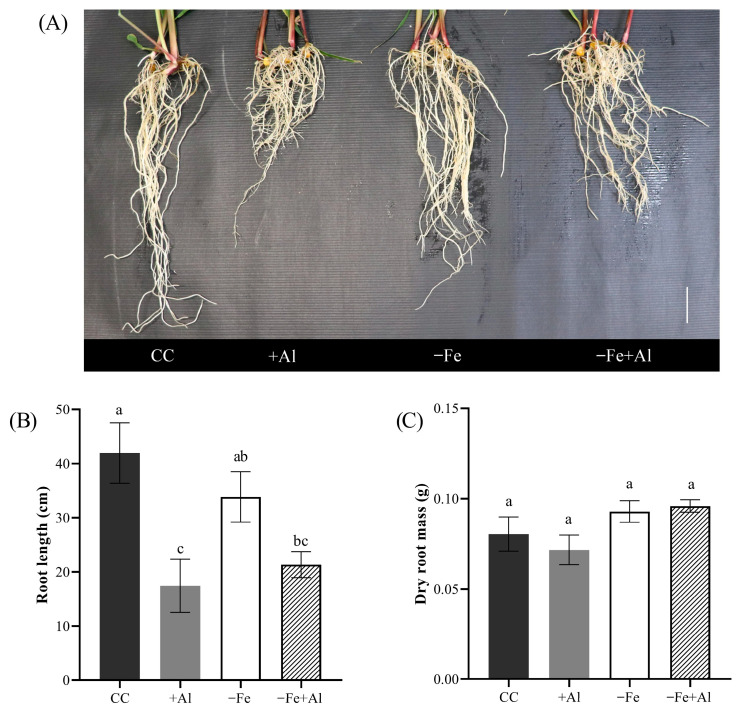
**Root phenotype of maize plants under Fe deficiency and Al excess.** (**A**) Representative images of root systems after nine days of treatment with control solution (CC), 300 µM AlCl_3_ (+Al), no Fe (−Fe), or combined (−Fe+Al). Scale bar = 5 cm. (**B**) Total root length and (**C**) root dry mass. Different letters indicate statistically significant differences according to Tukey’s test (*p* ≤ 0.05). Values represent mean ± SE (*n* = 4 for B; *n* = 7 for C).

To determine whether the Al-induced alleviation of Fe deficiency symptoms is conserved beyond the genus *Oryza*, we next evaluated its effect in maize under the same treatment conditions. Given that maize is more sensitive to Al compared to rice [19], we used 9 days of treatment instead of 21 days as with rice [51] and *O. rufipogon* (see above). As expected, root length was significantly affected by Al excess, as both +Al and −Fe+Al treatments resulted in reduced root length compared to the control (Figure 5A). Plants under −Fe also had a lower root length compared to the control but a higher one compared to +Al and −Fe+Al treatments (Figure 5B). These findings suggest that Al excess reduces root elongation independently of Fe deficiency. No statistical differences were observed in root dry mass among the treatments (Figure 5A,C). These findings indicate that overall biomass allocation to roots was maintained, even when Al-induced root shortening occurred.

### 2.6. Al Excess Alleviates Fe Deficiency Chlorosis in Maize

**Figure 6:**
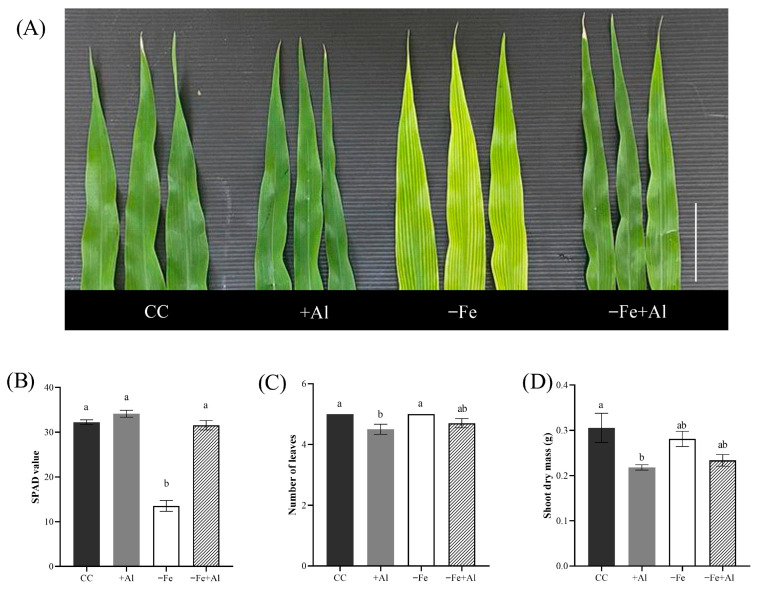
**Shoot phenotype of maize plants under Fe deficiency and Al excess.** (**A**) Representative images of the youngest fully expanded leaves after nine days of treatment with control solution (CC), 300 µM AlCl_3_ (+Al), no Fe (−Fe), or combined (−Fe+Al). Scale bar = 5 cm. (**B**) Relative chlorophyll content (SPAD values), (**C**) number of leaves, and (**D**) shoot dry mass. Different letters indicate statistically significant differences according to Tukey’s test (*p* ≤ 0.05). Values represent mean ± SE (*n* = 5 for B; *n* = 10 for C; *n* = 7 for D).

We next evaluated the effects of Al excess on leaf chlorosis. Although +Al treatment alone did not induce chlorosis, leaves were noticeably smaller than those of control plants. Leaves of Fe-deficient plants showed characteristic chlorosis, whereas −Fe+Al plants did not exhibit chlorosis, resembling the control and +Al treatments. To quantify the chlorosis alleviation effect, we measured chlorophyll content (SPAD values) in leaves. Plants grown under control, +Al, and −Fe+Al conditions showed similar SPAD values, indicating higher chlorophyll content and the absence of chlorosis. In contrast, plants under −Fe exhibited significantly lower SPAD values, confirming the observed chlorotic phenotype (Figure 6A,B). These observations demonstrate that Al excess mitigates chlorosis under Fe deficiency (Figure 6A), as also observed in *O. sativa* [51] and *O. rufipogon*.

Interestingly, leaves appeared slightly smaller when Al was added. Indeed, the +Al treatment resulted in a significant reduction in both leaf number and shoot dry mass compared to the control. While leaf numbers in plants grown in +Al or combined −Fe+Al were similar to the control but higher compared to −Fe, shoot dry mass was not statistically different compared to −Fe (Figure 6C,D). These data suggest a high sensitivity to Al in maize shoots.

### 2.7. Al Excess Mitigates Fe Deficiency-Induced Reduction in Photosynthesis in Maize

**Figure 7:**
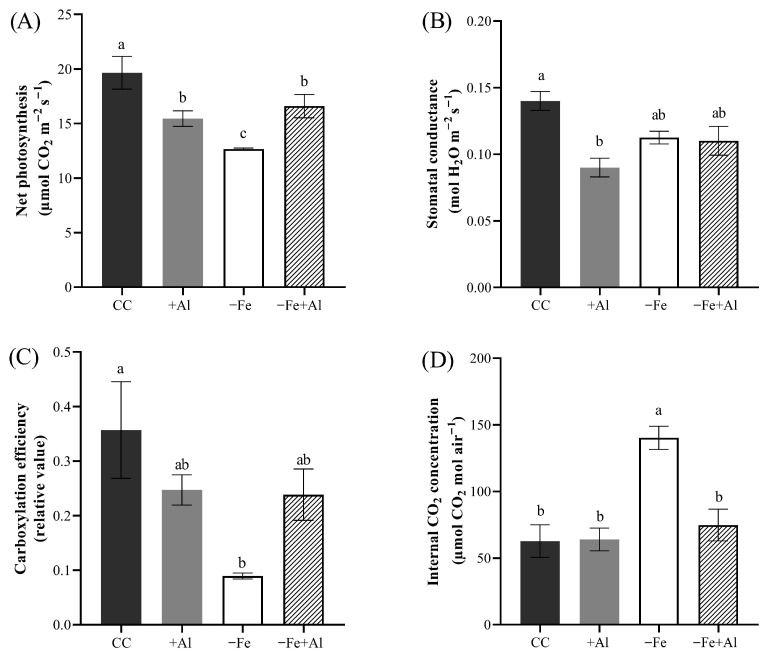
**Photosynthetic performance of maize plants under Fe deficiency and Al excess.** (**A**) Net photosynthesis, (**B**) stomatal conductance, (**C**) intercellular CO_2_ concentration, and (**D**) carboxylation efficiency in the youngest fully expanded leaf after nine days of treatment with control solution (CC), 300 µM AlCl_3_ (+Al), no Fe (−Fe), or combined (−Fe+Al). Different letters indicate statistically significant differences according to Tukey’s test (*p* ≤ 0.05). Values represent mean ± SE (*n* = 4).

Next, we investigated how gas exchange parameters were affected in maize, a C_4_ species. After six days of treatment, we measured net photosynthesis, stomatal conductance, internal CO_2_ concentration, and estimated carboxylation efficiency. As expected, plants under −Fe treatment exhibited a marked reduction in net photosynthesis compared to the control. Notably, −Fe+Al plants showed net photosynthesis levels significantly higher than those observed under −Fe alone, although lower than control plants (Figure 7A). Net photosynthesis in +Al plants was lower than control plants, but comparable to both −Fe and −Fe+Al plants (Figure 1A). These results suggest that Al excess partially alleviates the negative impact of Fe deficiency on photosynthetic activity (Figure 7A).

Stomatal conductance was significantly reduced in +Al plants compared to the control, whereas −Fe and −Fe+Al treatments showed intermediate values that did not differ significantly from either the control or +Al treatments (Figure 7B). Carboxylation efficiency was significantly reduced in −Fe plants compared to the control. In contrast, plants grown under +Al and −Fe+Al treatments did not differ statistically from the control (Figure 7C). Plants under −Fe accumulated a significantly higher internal CO_2_ concentration than all other treatments, indicating impaired carbon fixation. +Al and −Fe+Al treatments exhibited significantly lower internal CO_2_ concentrations than −Fe plants, comparable to those of control plants (Figure 7C), indicating that Al supply under Fe deficiency restored CO_2_ assimilation capacity. Altogether, these findings suggest that while Al does not fully recover all photosynthetic parameters under Fe deficiency, it substantially alleviates the detrimental effects of Fe deficiency on photosynthetic performance.

### 2.8. Al Excess Decreases the Expression of Fe Deficiency Marker Genes in Maize Roots

**Figure 8:**
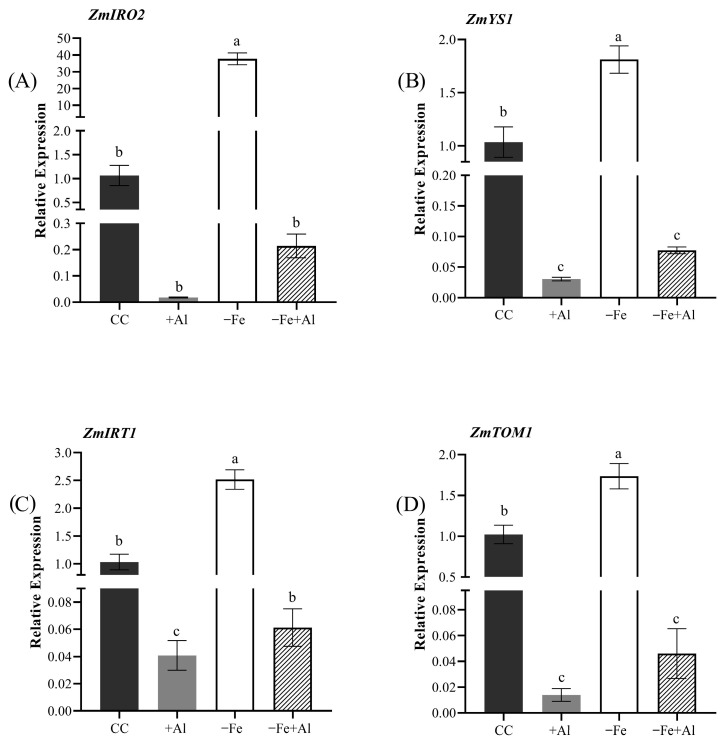
**Relative expression of Fe deficiency marker genes in maize roots under Fe deficiency and Al excess.** Relative expression of (**A**) *ZmIRO2*, (**B**) *ZmYS1*, (**C**) *ZmIRT1*, and (**D**) *ZmTOM1* in roots of maize plants treated for nine days with control solution (CC), 300 µM AlCl_3_ (+Al), no Fe (−Fe), or combined (−Fe+Al). Data were normalized to *ZmUBQ*. Different letters indicate statistically significant differences according to Tukey’s test (*p* ≤ 0.05). Values represent mean ± SE (*n* = 4).

To determine whether the chlorosis-alleviating effect of Al in maize is associated with changes in Fe deficiency response at the molecular level in roots, we evaluated the expression of four Fe deficiency marker genes, *ZmIRO2*, *ZmYS1*, *ZmIRT1*, and *ZmTOM1*, based on a previous work [43]. As expected, Fe deficiency induced the expression of all four genes compared to control conditions (Figure 8A–D). However, Al strongly inhibits expression of Fe deficiency-induced genes. Under +Al and combined −Fe+Al treatment, expression of all genes was markedly reduced compared to both controls and −Fe alone (Figure 8A–D). These findings suggest that Al exposure strongly represses Fe deficiency marker genes in maize.

## 3. Discussion

Although Al is generally considered toxic, several studies have reported possible beneficial effects in plants, which depend on the concentration and species [53,54,55]; however, no clear physiological role has been established to explains these responses. Recent work by our group demonstrated that Al alleviates Fe deficiency chlorosis in rice plants, likely through the induction of phosphorus (P) deficiency [51]. P deficiency is known to also alleviate chlorosis in several plant species [47,48,49,50]. However, it remains unclear whether the effect of Al on Fe deficiency is restricted to rice or also occurs in other Poaceae species. Here we demonstrate that this phenomenon is conserved in both the closest wild relative of rice, *O. rufipogon*, and in the C_4_ crop maize.

Fe is an essential nutrient for plants, directly involved in N and S assimilation, chlorophyll biosynthesis, and central C metabolism, including photosynthesis and respiration [16,56]. When plants such as cultivated and wild rice or maize experience Fe deficiency, they typically exhibit chlorosis, characterized by leaf yellowing, as a classical symptom [43,57]. In response to Fe deficiency, plants induce a set of root-expressed genes responsible for Fe uptake from the soil. These genes correspond to two distinct uptake mechanisms: Strategy I is characterized by the induction of *IRT1 (Iron-Regulated Transporter 1)* expression, and Strategy II is marked by the induction of *YSL15* expression [39,40,43]. Exceptionally, *O. sativa* and *O. rufipogon* employ a combined strategy, simultaneously inducing both *IRT1* and *YSL15* expression [43], as also observed under our experimental conditions (Figure 4). However, when Fe deficiency occurs together with Al excess, the transcriptional induction typically triggered by Fe deficiency is suppressed, resulting in expression levels similar to those in control plants. This indicates that Al interferes with the Fe deficiency signaling pathway by inhibiting the activation of Fe uptake genes. A similar response was observed in maize (Figure 8). These findings are consistent with our previous study in cultivated rice (*O. sativa*), which demonstrated that Al toxicity suppresses the induction of Fe deficiency marker genes to control-like levels [51], suggesting that this regulatory mechanism is conserved in at least three species of the Poaceae family.

*O. rufipogon* is the ancestral species from which *O. sativa* ssp. *japonica* was domesticated [3,52]. This wild relative represents an important reservoir of genetic variation for *O. sativa*, particularly for stress- and nutrition-related traits such as salt tolerance [58] and P deficiency tolerance [59], suggesting potential differences in ionomic regulation. Like cultivated rice, *O. rufipogon* is a semi-aquatic species that grows in permanently or seasonally flooded swamps and marshes, likely adapted to environmental conditions similar to those used for rice cultivation. Under such conditions, Fe^3+^ is reduced to Fe^2+^, increasing its bioavailability [60,61]. Likewise, Al^3+^ and PO_4_^3-^ ions also become more soluble and bioavailable in these environments [62]. Given the close phylogenetic relationship between the two species, and their shared ecological niches, it would be expected that the effect of Al on Fe deficiency chlorosis would be similar in *O. rufipogon* and *O. sativa*. Indeed, our data confirm that the Al-induced alleviation of Fe deficiency is not restricted to cultivated rice and therefore not a consequence of domestication. Nevertheless, environmental factors may strongly influence this mechanism, and whether other *Oryza* species or ecotypes not adapted to waterlogged conditions exhibit a similar response remains an open question.

Maize, on the other hand, is a domesticated crop characterized by C_4_ metabolism. C_4_ plants are known to exhibit distinct nutritional profiles, including higher N use efficiency compared to C_3_ and C_3_-C_4_ intermediate species [63,64], greater demand for reduced S forms [65,66], and altered P partitioning [67]. The homeostasis of N and P is closely linked to that of Fe [16], and Al and P share partially overlapping responsive genes networks [68]. However, it remains unknown whether the evolutionary transition from C_3_ to C_4_ metabolism also alters Fe homeostasis. Cultivated rice and its close relatives within the *Oryza* AA genome group, including *O. rufipogon*, employ a combined Fe acquisition strategy, whereas maize relies on the classic phytosiderophore-mediated, chelation-based strategy [43]. Our findings demonstrate that the Al-induced alleviation of Fe deficiency chlorosis also occurs in maize, a C_4_ species within the Poaceae family, suggesting that the mechanisms underlying this phenomenon might be conserved across the family. This conservation appears to persist despite the physiological and nutritional modifications associated with C_4_ metabolism, speciation, and domestication in maize. Moreover, considering that maize is substantially more sensitive to Al toxicity than rice [19,69], the observed alleviating effect of Al is unlikely to depend on intrinsic Al tolerance.

As previously noted, Fe is essential for photosynthesis, whereas plant exposure to Al is typically associated with increased production of reactive oxygen species and detrimental effects on photosynthetic performance. These adverse effects are often evidenced by reductions in stomatal conductance and in the activity of enzymes involved in carbon metabolism [70,71]. Although maize is generally more sensitive to Al toxicity than rice, under our experimental conditions, the negative impact of Al on photosynthesis was considerably less severe than that caused by Fe deficiency (Figure 7). This milder effect may reflect the modulation on the plant’s response to Fe deficiency, including the repression of genes associated with Fe uptake from the soil solution (Figure 8), resulting in a less pronounced deficiency response when Fe limitation occurs in the presence of Al. Additionally, it is noteworthy that Al toxicity exerted a stronger effect on root length and growth than Fe deficiency alone (Figure 5).

To deepen our understanding of how Al alleviates Fe deficiency symptoms in plants, future studies should dissect both the local and systemic mechanisms involved. Importantly, it is not clear whether this phenomenon is conserved in all Poaceae, given that we have tested only three species so far. Still, the fact that the same phenotype is found in two *Oryza* species and maize allows us to speculate that it might be conserved in other Poaceae. Moreover, the mechanism by which that occurs is not yet understood. We should explore whether P deficiency is induced by Al, how that alleviates chlorosis, and how elemental concentrations are altered in plants other than cultivated rice. Interestingly, P deficiency does alleviate Fe deficiency chlorosis in several species [46,47,48,49,50], suggesting that this could be conserved more broadly. However, more evidence is needed to confirm these hypotheses. Moreover, the answers to whether Al has the same effect and whether it operates by similar pathways to the known combined P and Fe deficiency phenotype are also not clear.

It should be noted that in the present study on *O. rufipogon* and maize, Fe, P, and Al concentrations or their localization were not measured. Consequently, we cannot determine whether the same P-related mechanisms previously proposed for cultivated rice also operate in these species. The evidence for Al-mediated alleviation of Fe deficiency presented here is based on (i) visual symptoms and chlorophyll/SPAD measurements, (ii) expression of Fe deficiency marker genes in roots, and (iii) photosynthetic performance in maize, rather than on direct determinations of Fe or P status. Elemental mapping (μXRF, LA-ICP-MS) and isotope tracing could reveal how Al influences Fe and P distribution and speciation, while split-root and mutant assays targeting Fe- and P-homeostasis genes would help identify key regulatory nodes. Integrating these approaches with chloroplast-level proteomic and physiological analyses will be essential to uncover how Al modifies Fe metabolism and photosynthetic function. Ultimately, expanding our investigations to additional Poaceae members and other botanical families will reveal whether the Al effect on Fe deficiency is conserved, as well as how ecologically relevant this interaction truly is.

## 4. Materials and Methods

### 4.1. Plant Materials and Growth Conditions

Experiments were performed in a controlled growth room at 26 °C ± 1 °C under a photoperiod of 16 h/8 h light/dark (150 μmol m^−2^ s^−1^), using a hydroponic system. Seeds of *O. rufipogon* (BRA 00004909-8) and Biomatrix hybrid maize (VTPRO3) were surface-sterilized by immersion in distilled water, sodium hypochlorite, and liquid detergent to minimize contamination and subsequently germinated in the dark at 25 °C for 24 h. *O. rufipogon* seeds were maintained for six days in Petri dishes with filter paper soaked in distilled water at 25 °C, while maize seeds were germinated for five days in gerbox^®^ with filter paper moistened under the same conditions. After germination, seedlings were transferred to vermiculite and cultivated in nutrient solution, as previously described [43], with slight modifications, detailed below [51]. Briefly, the solution consisted of 700 μM K_2_SO_4_, 100 μM KCl, 100 μM KH_2_PO_4_, 2 mM Ca(NO_3_)_2_, 500 μM MgSO_4_, 10 μM H_3_BO_3_, 0.5 μM MnSO_4_, 0.5 μM ZnSO_4_, 0.2 μM CuSO_4_, 0.01 μM (NH_4_)_6_Mo_7_O_24_, and 100 μM Fe^3+^-EDTA, with a pH adjusted to 4.5. When the third leaf was fully expanded, *O. rufipogon* plants were transferred to 2 L plastic pots containing nutritive solution, while maize plants were transferred to 2 L plastic pots containing nutritive solution aerated by air pumps.

After an acclimation period of nine days for *O. rufipogon* and three days for maize, plants were subjected to the following treatments: control, aluminum (Al) stress (+Al; 300 μM AlCl_3_ added to the nutrient solution), Fe deficiency (−Fe; no Fe added), and combined (−Fe+Al). Nutrient solutions were changed every three days. The wild rice experiment lasted 21 days, whereas the maize experiment lasted 9 days, given that maize growth is faster in our conditions. We established the optimal treatment length in pilot experiments. The aluminum treatment concentration was the same as that used for rice [51], allowing direct comparison.

All experiments were conducted at least twice, and one representative experiment with independent biological replicates is shown.

### 4.2. Growth Measurements

After 21 days of treatment for *O. rufipogon* and 9 days for maize, shoots and roots of five plants per species and per treatment were collected and oven-dried at 65 °C until constant mass was achieved. Dry mass was determined using a precision balance. For *O. rufipogon*, culm height was measured with a millimeter ruler. Root samples were placed in a thin layer (0.5 cm) of distilled water and scanned using an EPSON 11,000 scanner at 600 dpi resolution. The total root system length and leaf area were analyzed using WinRHIZO© Pro 2007 software [72]. For maize, root length was measured manually with a millimeter ruler after nine days of treatment.

### 4.3. Chlorophyll Measurements

For *O. rufipogon*, shoot samples from plants treated for 21 days were collected and immediately frozen in liquid nitrogen. Each sample consisted of a pool of seven plants, with four biological replicates per treatment. Samples were ground in liquid nitrogen to quantify photosynthetic pigments. Chlorophyll *a* and *b* concentrations were determined according to Hiscox and Israelstan [73], using the equations described by Lichtenthaler [74]. For maize, after nine days of treatment, relative chlorophyll content was measured using a SPAD-502 chlorophyll meter (Minolta, Tokyo, Japan; *n* = 5). The readings were taken in the final third of each leaf, and each sample value represents the average of three measurements.

### 4.4. Gene Expression Analyses

For RT-qPCR analyses, *O. rufipogon* plants were treated for ten days and maize plants for six days before roots samples (*n* = 4) were collected. Gene expression analyses were performed as previously described [43]. Root samples were pulverized in liquid nitrogen, and total RNA was extracted using Plant RNA Reagent (Invitrogen^®^, Waltham, MA, USA) following the manufacturer’s instructions. RNA quantity and purity were assessed using a Nanodrop^®^ spectrophotometer. First-strand cDNA was synthesized using M-MLV Reverse Transcriptase (Invitrogen^®^) after DNAse I (Invitrogen^®^) treatment, according to the manufacturer’s instructions. Relative gene expression was determined using a StepOne Real-Time PCR System (AppliedBiosystems^®^, Foster City, CA, USA), and data were analyzed following the method described by [75]. The *OsUBQ5* gene [76] was used as an internal reference for *O. rufipogon*, whereas *ZmUBQ* (*Zm00001d053834*) served as the normalizer for maize. The primer sequences for all analyzed genes are listed in Supplementary Table S1.

### 4.5. Determination of Gas Exchange Parameters

Net photosynthesis, stomatal conductance, and intercellular CO_2_ concentration were measured using a portable infrared gas analyzer (IRGA) system equipped with a red–blue LED light source and a leaf chamber (LCpro-SD, ADC BioScientific, Hoddesdon, UK). Measurements were performed on the youngest fully expanded leaf, with each reading recorded over a 15 min period (*n* = 4). During gas exchange measurements, the chamber conditions were maintained at 1000 µmol m^−2^ s^−1^ photosynthetic photon flux density (PPFD), 420 ppm CO_2_ concentration, 28 °C temperature, and ambient relative humidity (≈75%). The carboxylation efficiency was calculated as the ratio of net photosynthesis (A) to intercellular CO_2_ concentration (Ci).

### 4.6. Statistical Analyses

Data were tested for normality and homoscedasticity and analyzed by two-way analysis of variance (ANOVA), followed by the Tukey HSD post hoc test (*p* < 0.05), using SISVAR software (version 5.6) [77].

## 5. Conclusions

Al alleviates Fe deficiency chlorosis in both the wild rice ancestor *O. rufipogon* and the C_4_ crop maize, indicating that this capacity is conserved in these two species in the Poaceae family.

## Data Availability

The original contributions presented in this study are included in the article/Supplementary Material. Further inquiries can be directed to the corresponding author.

## Supplementary Materials

The following supporting information can be downloaded at https://www.mdpi.com/article/10.3390/plants15010159/s1, Table S1: Primer sequences used in this study.
