# Supplementary material for: Aluminum Alleviation of Iron Deficiency Chlorosis Is Conserved in Wild Rice Relative Oryza rufipogon and in Maize
Source: Plants (Basel). 2026 Jan 5;15(1):159. doi: 10.3390/plants15010159 (PMC12787346; doi:10.3390/plants15010159)
Supplement: Supplementary file 1 [file plants-15-00159-s001.zip › plants-4006699-supplementary.pdf]

Supplementary Table S1. Primer sequences used in this study.

**Locus**	**Gene name**	**Forward Primer 5’ → 3’**	**Reverse Primer 5’ → 3’**
OS01G0328400	*UBQ5*	ACCACTTCGACCGCCACTACT	ACGCCTAAGCCTGCTGGTT
ORUFI03G29910	*IRT1*	ACTGGTGCCCATTCTGC	GCGAGGATGGGGATGG
ORUFI02G27480	*YSL15*	GGTGCGGGGATGATTTG	CCATACAAACTTGTCATGCTG
Zm00001d053834	*UBQ*	CGGCAAGCAGCTGGAG	GCGCAGCACCAGGTG
Zm00001d042062	*IRO2*	GCCTCAACGACACGGAG	CCCAAAGGTGGAGTAGGTT
Zm00001d017429	*YS1*	GTCTGGGAGAAGGTGAACAG	GGCCAGAGCGAGAATGG
Zm00001d033446	*IRT1*	GCTTCTACAACCGGAGGAAG	GTCCTCGGGCTTGTCAG
Zm00001d041111	*TOM1*	CTTTCCTTGTGGACCGTAAGT	ACAAATGAACATACCTGCAATCG
